# Supplementary material for: Data-driven scenario-based model projections and management of the May 2021 COVID-19 resurgence in India
Source: PLOS Glob Public Health. 2022 Dec 13;2(12):e0001382. doi: 10.1371/journal.pgph.0001382 (PMC10021811; doi:10.1371/journal.pgph.0001382)
Supplement: S1 Table — (DOCX) [file pgph.0001382.s001.docx]

**Supporting Information for Data-driven scenario-based model projections and management of the May 2021 COVID-19 resurgence in India**

Edwin Michael^1,*^, Ken Newcomb^1^ and Anuj Mubayi^2,3^

^1^Global Health Infectious Disease Research, University of South Florida,

Tampa, FL, USA;

^2^ PRECISIONheor, Los Angeles, CA, USA;

^3^ Center for Collaborative Studies in Mathematical Biology, Illinois State University, Normal, IL, USA;

^*^ Corresponding author

Edwin Michael

Email: emichael443@usf.edu

S1 Table. A summary of the social measures applied by each Indian state from May 2021 – August 2021.

| State | Population | Social Measures Start | Social Measures Ending | Social Measures Used |
| --- | --- | --- | --- | --- |
| Uttar Pradesh | **199 million** | **April 29^th^** | **Complete lockdown ending May 30^th^, while night and weekend curfews persist until August 20^th^** | **Complete lockdown, curfews** |
| Maharashtra | **112 million** | **April 13^th^** | **Some restrictions are relaxed on June 17^th^, but restarts total lockdown on July 6^th^ . Social measure relaxations are announced on August 9^th^.** | **Complete lockdown: Individuals are not permitted in public places, and all non-essential businesses are shutdown, in addition to curfews** |
| Bihar | **104 million** | **April 19^th^** | **Relaxations start on June 8^th^, but various social measures persist through July 5^th^, such as only 50% attendance allowed in universities and schools.** | **Lockdown, curfews** |
| West Bengal | **91 million** | **May 1^st^** | **On June 14^th^, the state switches to partial lockdown, and curfews persist until August 30th** | **Lockdown/closure of all businesses,** |
| Madhya Pradesh | **72 Million** | **April 8^th^** | **Relaxations start on June 15^th^. Curfews and capacity restrictions in businesses persist into late July.** | **Lockdown in all urban areas of the state.** |
| Tamil Nadu | **72 Million** | **April 8^th^** | **Social measures persist into September 2021.** | **Complete lockdown, with gradual relaxations.** |
| Rajasthan | **68 Million** | **April 5^th^** | **Lockdown release starts on June 26, 2021. Business closures and curfews persist into July.** | **Initially, there were curfews and closure of businesses. In May 2021, total lockdown was enforced.** |
| Karnataka | **61 Million** | **April 2^nd^** | **Social measures are relaxed in a phased manner starting June 14^th^.** | **Lockdowns and curfews. Individuals entering the state had to show a negative COVID-19 test.** |
| Gujarat | **60 Million** | **April 7^th^** | **Night curfews are relaxed in some cities on May 26^th^. Night curfews are reinstated in late June amid a spike in cases, and last into September.** | **Night curfews** |
| Andhra Pradesh | **49 Million** | **May 3^rd^** | **July 5^th^** | **Mandatory quarantine for those entering the state, night curfews** |
| Odisha | **41 Million** | **May 2^nd^** | **June 17^th^** | **Lockdown, mandatory negative test for incoming travelers,** |
| Telangana | **35 Million** | **April 20^th^** | **Total lockdown ends June 20^th^, while night curfews and lockdowns in some villages persist into July.** | **Night curfew, lockdown** |
| Kerala | **33 Million** | **April 12^th^** | **On June 24^th^, the Kerala government announced lockdown relaxations, but short-term lockdowns and curfews will continue to be applied throughout June and July 2021.** | **Initially, only certain businesses were shutdown and curfews were applied. Kerala eventually applied a full lockdown.** |
| Jharkhand | **32 Million** | **May 13^th^** | **Relaxations start on June 30^th^, but many restrictions are extended into July.** | **Mandatory quarantine for visitors and restrictions on the hours of business.** |
| Assam | **31 Million** | **May 1^st^** | **August 16^th^** | **Curfews were applied in May, which turned into a total lockdown in early June. Lockdowns continue into late July.** |
| Punjab | **27 Million** | **April 7^th^** | **Relaxations of lockdown start on June 16^th^, while many restrictions apply until September 2021.** | **Night curfews are applied in April, which turns into a lockdown of all non-essential shops in May.** |
| Chhattisgarh | **25 Million** | **April 7^th^** | **April 19^th^** | **Complete lockdown** |
| Haryana | **25 Million** | **April 4^th^** | **Relaxations start June 6^th^, but Haryana continued to issue COVID-19 lockdown guidelines in early July.** | **Night curfew, followed by a complete lockdown in May.** |
| NCT of Delhi | **17 Million** | **April 14^th^** | **Opening of businesses occurs on June 13^th^, while restrictions are continued throughout July.** | **Night curfew, followed by a complete lockdown in early May.** |
| Jammu and Kashmir | **12 Million** | **May 9^th^** | **N/A** | **Curfews.** |
| Uttarakhand | **10 Million** | **April 21^st^** | **Relaxations begin on June 14^th^, while restrictions on business and curfews persist from July through September.** | **In April, a curfew was imposed, and in May, nonessential shops were ordered to shut down.** |
| Himachal Pradesh | **6.8 Million** | **April 11^th^** | **Relaxations begin on May 28^th^** | **Travel restrictions, curfews.** |
| Tripura | **3.6 Million** | **May 3^rd^** | **May 26^th^** | **Curfews** |
| Meghalaya | **2.9 Million** | **May 28^th^** | **June 7^th^** | **Lockdown** |
| Manipur | **2.5 Million** | **N/A** | **July 27^th^** | **Curfews** |
| Nagaland | **1.9 Million** | **N/A`** | **July 8^th^** | **Lockdown** |
| Goa | **1.4 Million** | **April 21^st^** | **Relaxations begin June 12^th^, while curfews persist from June through September.** | **Curfews, tourism restrictions** |
| Arunachal Pradesh | **1.3 Million** | **N/A** | **N/A** | **Lockdown** |
| Puducherry | **1.2 Million** | **N/A** | **N/A** | **Lockdown** |
| Mizoram | **1.0 Million** | **May 8^th^** | **August 7^th^** | **Lockdown** |
| Chandigarh | **1.0 Million** | **April 7^th^** | **Easing of restrictions is done in phases, from late May to July.** | **Lockdown, curfew** |
| Sikkim | **610,577** | **N/A** | **N/A** | **Lockdown** |
| Dadra and Nagar Haveli and Daman and Diu | **585,764** | **N/A** | **N/A** | **N/A** |
| Andaman and Nicobar Islands | **380,581** | **N/A** | **N/A** | **N/A** |
| Ladakh | **274,000** | **N/A** | **N/A** | **N/A** |
| Lakshadweep | **64,473** | **N/A** | **N/A** | **N/A** |

References: COVID-19 Notifications from KPMG. <https://home.kpmg/in/en/home/insights/2021/04/covid-19-notifications-april-2021.html>

<https://home.kpmg/in/en/home/insights/2021/05/covid-19-notifications-may-2021.html>

<https://home.kpmg/in/en/home/insights/2021/06/covid-19-notifications-june-2021.html>

<https://home.kpmg/in/en/home/insights/2021/07/covid-19-notifications-july-2021.html>

<https://home.kpmg/in/en/home/insights/2021/08/covid-19-notifications-august-2021.html>

<https://home.kpmg/in/en/home/insights/2021/09/covid-19-notifications-september-2021.html>
