# Supplementary material for: Data-driven scenario-based model projections and management of the May 2021 COVID-19 resurgence in India
Source: PLOS Glob Public Health. 2022 Dec 13;2(12):e0001382. doi: 10.1371/journal.pgph.0001382 (PMC10021811; doi:10.1371/journal.pgph.0001382)
Supplement: Attachment — Submitted filename: COVID_India_Reviewer_Responses_Final.docx [file pgph.0001382.s002.docx]

We thank both reviewers for their time and effort in reviewing our manuscript. Our response to each comment is given below, along with the line numbers where changes to the manuscript were made.

**Responses to Reviewers**

Reviewer #1:

Line 79 reference missing

**Thank you. We have added the missing reference (Line 80)**

Line 90. It would really help the reader understand the importance of this paper if there was a summary, probably here in the introduction of the actual measures implemented in India as a whole, and what happened in individual states. I am having problems matching actual lockdown periods with the modelling. For example, wasn’t a lockdown was imposed in Maharashtra on the 5th April and many states had stated lifting lockdown by mid-June? This does not match the timing in Fig 4.

**We have added a summary of India’s lockdown policies in 2021 to the manuscript (Lines 91-95). While there were some restrictions in Maharashtra that were imposed in early April, there were no nation-wide lockdowns in place. In early May 2021, many Indian states were imposing strict lockdowns, which is consistent with our simulations.**

Line 102. The model makes some critical simplifying assumptions. Whether these makes an overall difference is unclear but should be addressed in the methods or discussion or both.

As far as I can tell from the ODEs at https://github.com/EdwinMichaelLab/COVID-SEIR-India the vaccine is “all or nothing” I.e. there is a certain probability that a vaccinated person is 100% immune from infection or has no immunity at all. This is a critical factor. Data from the UK, and other countries (including India) mainly obtained with the wild type and Beta VOC show that vaccinated people who become infected are significantly less likely to transmit (so the vaccine is leaky) and are much less likely to require hospitalized. This model does not reflect this reality.

Model makes no distinction on the contribution to the community force of infection for people in isolation, in hospital or in an ICU. Again, I can’t tell if this is critical or not, but goes strongly against public health practices. If this was the case, then there would be no point in isolating individuals who become infected.

I don’t see any delays built into the ODE to account for the delays in acquiring immunity following vaccination.

**Indeed, our model makes simplifying assumptions as all models do to the extent of knowledge available at the time of modelling. In the ODEs for predicting the impact of vaccination, we move individuals from the susceptible state (S) to the vaccinated state (V) directly according to daily vaccination data. This means we are treating people as either in the vaccinated class or are not in other words. However, vaccinated individuals are not fully protected – they can be reinfected at a slower rate, given by the factor of (1 - vaccine_efficacy) as captured in the equation for V (https://github.com/EdwinMichaelLab/COVID-SEIR-India). After infection, we do treat vaccinated and non-vaccinated individuals the same, plus do not include delays in acquiring immunity following vaccination. We did this to constrain the number of estimated parameters, using adjustments made to the rest of the parameters during sequential calibration to match the observed data sufficiently well. Note the model manages to capture improvements in hospital outcomes over time via the gamma (recovery rate) parameters, meaning that we are able to reproduce population-level patterns reliably with the key parameters and structures included in the model, implying that many parameters may not be critical for this prediction purpose. Regarding contributions of individuals in lockdowns, hospitals and in ICU to transmission, we use reductions in the social contacts (reduction in d) to effectively remove individuals from contact other by various rates, whereas we allow individuals in hospitals and ICU to continue to transmit (to capture any possibility of transmitting infection to hospital personnel, visitors etc). We have included sentences regarding the simplifications made in the Methods (Lines 145-149)**

Line 106. Would be clearer if the actual dates for calibrating the model were quoted , Was this 21 Jan 2020 to 5 May 2021 or a shorter period? If it was up to 5th May 2021, then there seems to be a major discrepancy in the fit of the model to the actual data in early May 2021. By 5th May the infection had peaked (i.e. rate of increase was close to zero), whereas the data in Fig. 2,using median values for the model, suggests that the slope on the 5th May was close to maximal. This is critical for Fig. 4 when lockdowns are introduced. Please comment, especially as a model built on sets of differential equations is primarily modelling the slope of the epidemic.

**Thank you. Yes, we fit the model up to 5th May 2021, and had added this in the manuscript (under Sequential Model Calibration in Methods, line 111). As can be seen from Fig 2 the median model predictions closely matches data (red circles in Fig 2a). The median model projections after this date (black line) assumes that both the social distancing and vaccination rates up the data calibration point remain constant into the future. This therefore constitutes the expected pandemic growth in the absence of any further interventions, i.e. it provides the counterfactual situation. The lockdown scenario simulations shown in Figure 4 more closely represent what actually occurred in India; the model forecasts given in the figure, eg for a lockdown of 75% and a 30 -day also, demonstrates the ability of our model to capture what happened in India following the introduction of partial lockdowns after May 5^th^ 2021. We’ve amended Figure 4 (below) to show this effect of a severe 30-day lockdown, which closely fits the daily confirmed case data in India. We have discussed this fully (lines 188 to 206; lines 325 to 327).**

Line 133. Efficacy quoted for wild type is only if the second vaccination at 12 weeks. Second vaccination at 6 weeks gives much less efficacy. See https://doi.org/10.1016/S0140-6736(21)00432-3 for wild type. There is emerging data from the UK that for Delta, at least for non-geriatric populations, AZ efficacy against Delta is similarly reduced if the second vaccination is at 6 weeks. E.g., Fig 4 AZ 50-65 in <https://doi.org/10.1101/2021.07.26.21261140>

**This is a good point, thank you. We ran simulations showing the effect of using a lower efficacy against both the original and delta variants, when using a 6-week spacing between the 1st and 2nd doses. Counterintuitively, using a 6-week spacing results in a quicker uptake of the second dose, which balances the effect of a lower vaccine efficacy, and results in lower daily infections. As you can see from the figure below, the intensity of social measures has the greatest effect on the short-term trajectory, while the vaccine dose spacing has a less significant effect. This also shows that social measures rather than vaccine efficacy and spacing are greater drivers of the pandemic. We have previously shown this complex trade-offs between these measures in Young et al (https://arxiv.org/pdf/2103.06120.pdf).**

Line 147. Can the authors gives some concrete examples of what would be required to decreased by 25% or 50%. Without such calibration it is not possible to know whether this level of reduction is realistic.

**In early May 2021, half of the Indian states went under “partial lockdown”, while the rest imposed a “full lockdown”. The imposed lockdown measures varied from state to state, including shutting down public transport and closing restaurants. (**[**https://www.reuters.com/world/india/india-posts-record-daily-rise-covid-19-deaths-case-numbers-surge-2021-05-08/**](https://www.reuters.com/world/india/india-posts-record-daily-rise-covid-19-deaths-case-numbers-surge-2021-05-08/)**) Unfortunately, there isn’t a universally accepted definition of these two terms (i.e. partial versus full lockdown)** [**https://gh.bmj.com/content/5/10/e003319**](https://gh.bmj.com/content/5/10/e003319)**.**

**Here, we define mild, moderate and strong lockdowns as reducing social contacts estimated on May 5^th^ by 25%, 50% and 75% respectively from that day onwards. The model forecasts in Fig. 4 suggest that either very strong lockdown measures are needed to be taken for 30 days, or moderate lockdown measures for 60 days to match the data which transpired in India. Both of these scenarios fit the daily case data, shown in dark circles. We have also discussed this (lines 292-295)**

Line 165. Link to reference 22 is “broken”

**Thank you. We have corrected the link.**

Line 169. Fig. 1 Blue line shows data on first vaccines to 5/5/2021 and then the projection to the end of the year. However, this projection is a long way from the actual vaccination coverage that has been achieved to the 19th November. It is currently about 55% single dose – i.e., about twice the projection.

**We agree - we applied the average daily vaccination rate when simulating into the future, which underestimated the actual vaccination rate. For this reason, we ran scenarios with a biweekly increase in vaccination rate, which much more closely match the actual vaccination rate, as shown below. We now add this as a result (Fig 1) in the ms.**

Line 214. It is not clear from the description or the web page listing the ODE how independent estimates are made for d and β. As far as I can tell in the ODE, these parameters only occur as the product i.e., dβ, so I can’t see how these were separately estimated. Is there something I am missing? If so it would be useful to make this clear in the text. Confidence intervals and covariance of d & β?

**This is a good point. We start with a range of priors for d and beta, which were used in our previous work and are taken from literature. We use a wide range for d (0.2 to 0.9), reflecting our uncertainty in the strength and compliance of social measures. Given these priors, we simply used our calibration method to select the individual values in** dβ **that matches the data best.**

Line 241. What is shown is a correlation between the model prediction and observed cases. Correlation does not mean causality.

**Thank you. We’ve amended the text to avoid making such a strong assertion.**

Line 252. 1,470,000 vaccinations per day. Are these first vaccinations, or combined first and second vaccinations? Can these be specified separately.

**Thanks - that number refers to the first dose vaccinations. We’ve corrected the text to reflect this.**

Line 247. I suggest you do a reality check here on the modelling. During most of April the rate of increase in daily cases was indistinguishable from a true exponential increase with a rate constant of 0.069 per day. Depending on assumptions about the serial interval, that corresponds to an R of about 1.4. Since the % of people vaccinated only had a very minor change over April/May, this suggests that the transmission from person to person (presumably as modelled by the parameter “d”) would need to decrease by about 1 – 1/1.4 = 29% before the epidemic would flatten. *So a 25% decreased “d” would be expected to give a slow increase in daily infections* shown in Fig. 4 red dotted line for any of the 30, 60 or 90 day lockdown scenarios. On the other hand, a 50% decrease in “d” should lead to decreased infection rates (magenta dotted line) as shown. Is the discrepancy because the epidemic had already peak prior to the lockdowns modelled in Fig 4, in which case the 25% reduction may have been irrelevant?

**Any decrease in d (an increase in protection due to social measures) will cause decreased infection rates. In Figure 4, we show the effect of a 25% and 50% increase in social measures (25% - 50% decrease in the value of d). Both of these scenarios show a drop in infection rates for the duration of the lockdown modelled.**

Line 342. Reference missing

**Thank you - we’ve now included this reference.**

Discussion: This paper and the diagrams are essentially similar to the preprint posted on Research Square on the 3th June. I can’t see any appreciably new modelling since then. I understand PLOS encourages pre-prints but the authors now have the unfortunate situation where their projection can be tested against what controls were put in place, what vaccination levels have been obtained and the number of cases over the period June – November. History has not been kind. As far as I can see, what has actually happened in India does not closely match any of the scenarios/outcome in Fig 4. This largely invalidates at least the first and third goals in the Introduction, lines 85 to 87.

In a revision of the paper, I suggest re-running the model, without further calibration (i.e., use a database locked on the 5th May) against the scenario that actually took place taking into account different situations in different states. Then there can be a real test of the model and a test of the second goal in the Introduction “to facilitate insight into the underlying factors driving the wave of resurgent cases”

**The median model prediction, which assumes that social measures and vaccination will be constant into the future, shows a steady growth of cases after May 5th. However, the +50% increase in social measures scenario fits the data well. The figure given in the preprint is shown below - our model is fit to the green data points, while the blue data points are the latest case data, released after publication. The data follows the prediction of cases until the lockdown is released on June 5th. This shows that our predictions have support in data.**

Reviewer #2:

The manuscript is well written and an interesting piece.

**We appreciate the comments and positive feedback.**

Just a few concerns: 1. There is a mix of both past and present tenses, this should be corrected to past since it is a reported event/activity.

**Thank you - we’ve corrected the tenses to be consistent as best as possible, but given the length of time taken by the review process it is a challenge to address what was written in the heat of the 2^nd^ wave in India a year ago in the right tense!**

2. The abstract section does not clearly summarize the purpose of the study and the results of the study and methodology. More active words would help.

**Thanks - we’ve revised the abstract to better summarize the study.**

3. In the introduction section of the manuscript, Line 85, it would be more preferable to use "Objectives" i.e we had three objectives rather than "Goal".

4. In the discussion section, Line 404, it would be better to say " Finally we recommend that" rather than saying " we indicate in closing"

**We agree with the reviewer and have made the above changes to the manuscript.**
