# Supplementary material for: Data-driven scenario-based model projections and management of the May 2021 COVID-19 resurgence in India
Source: PLOS Glob Public Health. 2022 Dec 13;2(12):e0001382. doi: 10.1371/journal.pgph.0001382 (PMC10021811; doi:10.1371/journal.pgph.0001382)
Supplement: Attachment — Submitted filename: India Reviewer Responses_second_round 10.22.2022.docx [file pgph.0001382.s003.docx]

We thank the reviewer for his/her second set of comments on our manuscript. It has been a challenge to rewrite our ms in light of the comments made given the lapse of time since our first submission. Nonetheless, our response to each comment is given below, along with reference to where changes to the manuscript were made. Editor(s), please note that light of the reviewer’s comments, we have also made a change to the title of the ms, which we feel is now more aligned with the Reviewer’s sentiments. We have also revised the previous Figure 4 and have also included a new Figure 5 plus a new Table S1 as supporting Information.

We give our point-by-point response below in bold

**Reviewer #1:**

I’ve been through the revised MS and I note that many of the suggestions have been adopted. Thank you for that. However, there are still serious problems with this MS. Not withstanding the comments by the Authors on p 53 of the PDF, the model does NOT predict the course of the epidemic in India. As shown in the plots of actual cases in the graphs on pages 49 and 53 of the PDF, (and for a more extensive view see https://ourworldindata.org/coronavirus ), cases continued to fall after most states lifted restrictions on the 15th June 2021, reaching very low numbers at the end of December 2021. The model suggests that the restrictions imposed around the 5th May decreased “d” by about 50%, but then regardless of the assumptions about the rates of vaccination, there should have been an immediate and substantial increase in cases following the lifting of restrictions. This did not occur. Cases continued to fall with NO sign of any inflection in the course of the epidemic when the controls were lifted. Clearly the model fails this critical test and until or unless the model is reformulated to get a better match, then I am afraid I can’t trust any of the conclusions. On the assumption that the model can be re-worked to give something that matches what actually happened, there are some other points that authors may like to address. However, I was stopped by the lack of match between the model and what happened after June 15, and I have not been though all of the MS in detail again. There may be more points I have missed.

**Response: It is apparent that due to lack of clarity on our part the Reviewer may have misunderstood that our work is based on modelling scenarios to evaluate the likely future paths of the pandemic arising from implementing plausible interventions from May 5^th^ 2021. We pointed out the need for both projecting the size of the growing wave and for intervention modelling analyses to inform policies on how best to curve the rapidly expanding contagion in para 2 of the Introduction, but this rational for our work may not have registered fully. Modelling scenarios as the Reviewer will know is not meant to predict the future exactly but rather to explore and compare a range of different plausible futures and in our case the pathway (identified via comparison of forecasts of intervention scenarios) to get to the future of interest (in our case the curbing of the spread of the May wave). We apologize for this lack of clarity and have added text to the new Abstract, Introduction, Methods and Discussion sections to better explain our aims in this regard.**

**Nonetheless, we have now added case data through to the end of 2021 to demonstrate that our 60-day lockdown with 50% social measures and ramped up vaccination model can and did reproduce the aggregate infection data reported for India after May 2021 (see new Figure 4 comparing results from this scenario against data now added in the figure to end of 2021). So, although our aim as noted above, was not on accurate predictions, but focused on exploring trade-offs between lockdown versus vaccination scenarios for curbing the pandemic, this result supports the credibility of using our simulations for guiding policy and management decisions as it simulates approximately what did take place in terms of lockdowns/social restrictions and vaccination ramp ups in the country (see below). Note also we simply added the data from India to end of Dec 2021 for comparison with scenario predictions in Figure 4, ie we have not reworked or refitted our original model. This shows that our scenario-based modelling exercise and indeed our model parameterized with data to May 5ft 2021 and then used to forecast/project the impacts of various intervention scenarios can match evolving future data if drivers (lockdown durations and strength and vaccinations) are correctly included in the model. This highlights that such data-driven model-based scenario simulations, as formulated and carried out by us, contrary to Reviewer’s criticism, can provide a valuable informational tool to help guide real-world pandemic policy development.**

**The Reviewer also is fixed on his/her assertion that most states in India lifted restrictions on 15^th^ June 2021. As we show in new Table S1 this is simply not correct, with many states fully relaxing various social restrictions well into July and even into August 2021. This approximates the aggregate 60-day lockdown and 50% social measures that we modelled in this paper, which indeed forecasts the continuous decline in cases to end of 2021 without to use the Reviewer’s words any sign of inflection in cases (Figure 4). This is exactly in line with how the cases declined in India from May to end of 2021 (see figure below). In any case, to address the point about the lack of case resurgence forecasted by our model made by the Reviewer, our simulations of the 30-day lockdown scenario (Figure 4) clearly show that if restrictions were lifted after a month (roughly the June 15^th^ 2021 date that the Reviewer refers to) then the model does project a rise in cases depending on levels of social measures and vaccinations implemented. However, of course, this did not happen in India which our model results and the actual policies carried out in India indicate is because many states continued with social restrictions of different kinds well into July/some into August and vaccinations also ramped up from 1.1 million doses daily in April to > 4 million by end of the year (see figure below), which is approximated by our 60-day lockdown, 50% social measures and ramped up vaccination scenario. So, contrary to the Reviewer’s claim, predictions from this scenario does match the data remarkably well, and therefore our model has not failed the critical test that the Reviewer appears to have set for assessing the credibility of our work. This is even though we focused on simulating plausible aggregate-level future intervention scenarios and did not model the interventions that took place in India exactly after May 5^th^.**

**Finally, we would also like to emphasize the significance of our scenario-based modelling work for policy, viz. that while shorter and less intense lockdowns would result in a relatively larger increase in cases when lockdown measures are released, if a longer or more intense lockdown is applied (50% and above lockdown for 60 days or longer), there will be no rise in cases with cases subsequently declining over the long term. This is exactly what was observed in the data (see figure). While as noted above this outcome provides an positive assurance test for the performance of our model, our goal here is to demonstrate that when SEIR models are paired with plausible scenarios, reasonable forecasts can be achieved and can guide policy making. In this case, our results indicate that India needed to go into an immediate lockdown for at least 60 days, in order to arrest the emerging wave.**

1. To understand this model, it is critical to know what happened with regard to restrictions in India. There is still no where near enough detail in the Introduction to enable a reader to

match the modelling with what happened “on the ground”. For example, I don’t see

anywhere in this MS that says most states lifted their restrictions around June 15th 2021.

**Response: The reviewer must understand that at the time of modeling, we did not know the extent and timing of the social measures that would be applied by each state. This is why we employed a model-based scenario analysis to explore the impact of plausible lockdown and vaccination scenarios that could be followed by India to curb the rising wave. As noted above, in a scenario analysis, we try to forecast the impact of interventions that could be followed as opposed to predicting the outcomes of interventions that actually happened on the ground. But we agree absolutely that it will be useful to see if our scenario projections matched what happened in India. This is because from a safety engineering perspective, data generated by epidemiological models can be considered to be safety-critical, especially when it is to be used for policy-making, and therefore must hold up to the requirement of assured performance. We refer to our response above to highlight, in this regard, that our model forecasts for cases based on the 60-day lockdown/50% social measures/ramped up vaccination do match what happened on the ground in India post May 2021, thereby meeting this requirement. We have added a new section in Results to highlight this result in the 2^nd^ resubmission.**

**To the point made by the reviewer about lifting lockdowns on June 15th, the actual lifting of social measures varied from state to state. We have constructed a new Table S1 to summarize the social measures followed by Indian states, which highlights not only the variable measures used by the states but also the lengths of time these measures were implemented in each state. Even the government of India recommendation on June 29th was that states could reopen from then on in a calibrated manner, presumably by reacting to changes in local incidence. So, the reviewer is not correct in saying that most states lifted their restrictions around June 15^th^ – partial lockdowns and curfews were followed into July and even August. Therefore, we believe the scenario of the lockdown with at least 50% level of social measures along a 60-day (until July 4th 2021) duration of social measures actually do represent reasonably well what did take place in India on the aggregate. We include new text in the resubmission to highlight this matching of modelling results with data.**

2. The aims need to be revised. It is no longer relevant “to project the size and likely future

paths of the viral resurgence in India” . We know what happened. It may be appropriate to

expand on the second aim, to understand the relative importance of the various factors that

did result in the control of the May 2021 wave of SARS-CoV-2 infection. The discussion

likewise need to consider how well the model predicted outcomes as well as explain the

relative importance of different measures.

**Response: We have alluded to the need for this projection in both the abstract and in greater detail in the first 3 paragraphs of the Introduction, which the Reviewer appears to have missed or has not fully appreciated. As we note there one key information required by policy makers when dealing with a complex outbreak emergency is data on the likely size of a surge and its impacts so that rational decisions can be made on whether costly resources should be allocated to interventions. Providing the counterfactual in this case, especially in terms of the exponentially growing dynamics connected with the May wave, also is instructive as it additionally helps determine the speed with which action needs to be taken. Our projection of the size of the growth of the May wave if unchecked in this regard for example highlighted the policy need to immediately implement control to curb the rise in cases seen in May itself. Thus, providing both an assessment of the likely impact of an emergency (size of the wave in the present case and its health impacts) plus evaluation of responses on how best to manage the potentially catastrophic outcomes are two important arms of any emergency management cycle, a procedure which we follow and highlight in this paper. As we note at the end of para 3 in the Introduction, a key function of epidemic modelling is also to illustrate the nature of the exponential growth dynamics involved in epidemic spread to policy-makers, who otherwise can misread the magnitude of the challenge posed by a pandemic.**

**The Reviewer confuses the difference between model-based scenario analysis to explore future possibilities versus modelling the impacts of the actual interventions that took place with his/her insistence on asking us to use our results to understand the relative importance of factors that did result in the control of the May 2021 wave. We again reiterate that this is not the goal of this (or any scenario-based) study, viz the use of models for predicting the relative importance of different actually implemented measures. As noted above, scenario approaches are based on the idea that while the future is uncertain it can be knowable for a dynamical system by varying how its driving forces (in our case changes in interventions impacting transmission) may change or be changed over a future period of interest. Thus, our aim was to use plausible scenarios to identify the best set of interventions for curbing the May 2021 wave as fast as possible – this is a policy or programme design question for bringing about a beneficial future not an analysis of what actually took place on the ground in the future using models. This is what we meant in para 2 of the Introduction by stating that the 2^nd^ informational need for managing a pandemic are results of intervention modelling analyses (lines 55 – 62). We have included new text (last para) in the Discussion to highlight this difference connected with undertaking modelling futures using model-driven scenario analyses for proactively identifying interventions to manage the May wave as rapidly as possible, as opposed to reacting to data as the basis for curbing the wave.**

3. As pointed out by the other reviewer, there are still more places where the tense could be

modified. E.g., in the figure legends.

**Response: We’ve corrected the tense as best we could throughout the text. Once again, we ask the reviewer to understand that we were making future projections of the pandemic in late May 2021, and that this is not a retrospective analysis to use past tense throughout. We have however done our best to take account of this issue.**

4. Sorry to be a bit slow, but I’ve read the authors reply but still don’t understand how they

separately estimate β and d. As pointed out before they always occur as the product in

their ODE. Did the authors do this by estimating β separately first? On their github site they

define β = R0 * γ. There are multiple different γ depending on the path though the SEIR

model, but most paths have a γ of ~0.23. With a β of 0.325 that would make R0 about 1.4.

That is impossibly low. (And as far as I can tell, it has to really be R0, as Rt, the ongoing R, is a function of d and natural and vaccine induced efficacy, so to use Rt to calculate β gets to be a circular argument again) The original estimate of R0 for the Wuhan variant is about 4 and for Delta was a bit higher, in the range of 6-8 so there seems to be major problem here with the parameterization. (Incidentally and presumably not important for this paper, but on the authors’ github site, the efficacies following single or double vaccinations are apparently

reversed).

**To estimate beta and d in our simulations, we first went to the literature and found reasonable estimates for both of these quantities. We used those estimates to set the initial ranges of priors. Then, we use the best-fitting sets of parameters to produce forecasts. We have added text under Methods to clarify the procedure better in this 2nd resubmission.**

**As to defining beta, please note we do not define beta as R0 * γ, as the Reviewer wrongly presumes.** **This is the methodology used by one of the works we cite (and referenced in Github) and is definitely NOT the methodology we used in this paper. As noted above, we estimated beta directly from the data using our sequential Bayesian calibration approach (please see Methods).**
